# Complete chloroplast genomes of five *Cuscuta* species and their evolutionary significance in the *Cuscuta* genus

**DOI:** 10.1186/s12864-023-09427-w

**Published:** 2023-06-08

**Authors:** Hangkai Pan, Lyuben Zagorchev, Luxi Chen, Yutian Tao, Chaonan Cai, Ming Jiang, Zhongshuai Sun, Junmin Li

**Affiliations:** 1 Zhejiang Provincial Key Laboratory of Plant Evolutionary Ecology and Conservation, School of Life Sciences, Taizhou University, Taizhou, 318000 China; 2 School of Life Sciences, Shanghai Normal University, Shanghai, 200234 China; 3 Department of Biochemistry, Faculty of Biology, Sofia University “St. Kliment Ohridski”, 8 Dragan Tsankov Blvd., Sofia, 1164 Bulgaria; 4 School of Electronics and Information Engineering, Taizhou University, Taizhou, 318000 China; 5 School of Advanced Study, Taizhou University, Taizhou, 318000 China

**Keywords:** Chloroplast genome, *Cuscuta* spp., Phylogenetic analysis, Subgenus, Gene reduction

## Abstract

**Background:**

*Cuscuta*, a parasitic plant species in the Convolvulaceae family, grows in many countries and regions. However, the relationship between some species is still unclear. Therefore, more studies are needed to assess the variation of the chloroplast (cp) genome in *Cuscuta* species and their relationship with subgenera or sections, thus, providing important information on the evolution of *Cuscuta* species.

**Results:**

In the present study, we identified the whole cp genomes of *C. epithymum*, *C. europaea*, *C. gronovii*, *C. chinensis* and *C. japonica*, and then constructed a phylogenetic tree of 23 *Cuscuta* species based on the complete genome sequences and protein-coding genes. The complete cp genome sequences of *C. epithymum* and *C. europaea* were 96,292 and 97,661 bp long, respectively, and lacked an inverted repeat region. Most cp genomes of *Cuscuta* spp. have tetragonal and circular structures except for *C. epithymum*, *C. europaea, C. pedicellata* and *C. approximata*. Based on the number of genes and the structure of cp genome and the patterns of gene reduction, we found that *C. epithymum* and *C. europaea* belonged to subgenus *Cuscuta*. Most of the cp genomes of the 23 *Cuscuta* species had single nucleotide repeats of A and T. The inverted repeat region boundaries among species were similar in the same subgenera. Several cp genes were lost. In addition, the numbers and types of the lost genes in the same subgenus were similar. Most of the lost genes were related to photosynthesis (*ndh*, *rpo*, *psa*, *psb*, *pet*, and *rbcL*), which could have gradually caused the plants to lose the ability to photosynthesize.

**Conclusion:**

Our results enrich the data on cp. genomes of genus *Cuscuta*. This study provides new insights into understanding the phylogenetic relationships and variations in the cp genome of *Cuscuta* species.

**Supplementary Information:**

The online version contains supplementary material available at 10.1186/s12864-023-09427-w.

## Background

Chloroplasts are essential in photosynthesis and carbon fixation and thus, promote plant growth and development [1]. Chloroplasts are highly conserved based on gene size, gene content, and sequence order. They comprise a single circular molecule with a quadripartite structure that harbors two copies of inverted repeats (IRs) that separate large and small single-copy (LSC and SSC) [2]. The cp genomes encode 110–130 genes that range from 100 to 180 kb in length [3] that are primarily associated with photosynthesis, transcription, and translation [2]. Since complete cp genome sequences are contained in a single plastid genome, they have recently become popular for plant species identification, taxonomy, and phylogenetic analyses [4].

The genus *Cuscuta* belongs to the Convolvulaceae family and has approximately 200 species that are widely distributed worldwide [5]. *Cuscuta* is a holoparasite and obtains nutrients, water, and organic compounds from the host via haustoria [6]. Engelmann (1859) divided 77 *Cuscuta* species into three groups based on the morphology of their stigma [7]. Yuncker (1932) also divided 158 *Cuscuta* species into the three subgenera *Cuscuta* (28 species), *Grammica* (121 species), and *Monogyna* (nine species) based on dehiscence of the fruits [8]. Revill (2005) indicated that the molecular phylogeny of 15 species of *Cuscuta* belonged to three subgenera based on three types of plastid DNA (*rbcL*, *rps2*, and *matK*) [9], consistent with the conclusions of Yuncker [8]. Garcia (2014) also divided 131 *Cuscuta* species into four subgenera (*Monogynella*, *Grammica*, *Pachystigma*, and *Cuscuta*) using *rbcL*, *nrLSU*, fruit cracking, style number, and stigma shape [5]. Garcia indicated that *Pachystigma* does not belong to the subgenus *Cuscuta* but is related to the subgenus *Grammica*, a conclusion that was inconsistent with those of Yuncker [8], Revill [9], and McNeal [10]. Costea et al. (2015) grouped 194 *Cuscuta* species into four subgenera (*Monogynella*, *Cuscuta*, *Pachystigma*, and *Grammica*) based on the morphological and biogeographical predictive value [11], which was consistent with the conclusions of Garcia [5]. Banerjee and Stefanović (2020) classified six *Cuscuta* species into four subgenera using the whole cp genome sequencing method [12], which was consistent with the previous phylogenetic relationship based on morphological [11] and DNA sequences [5]. However, Banerjee and Stefanović used few *Cuscuta* species [12]. Therefore, a precise phylogenetic relationship should be assessed by including more species of *Cuscuta*. Moreover, the phylogenetic analysis of particular *Cuscuta* species is necessary to clarify the phylogenetic location of each species. For example, the phylogenic location of *C. epilinum* has been inconsistent in different studies. Revill (2005) showed that *C. epilinum* belongs in the subgenus *Grammica* [9], while McNeal (2007) found that *C. epilinum* belongs in the subgenus *Cuscuta* based on the nuclear ribosomal internal transcribed spacer (*nrITS*) *rps2*, *rbcL*, and *matK* [10].

The cp genome encodes numerous structural proteins that are essential for photosynthesis. It also encodes ribosomal proteins and structural RNAs [13]. Therefore, the loss or mutation of genes in chloroplasts could affect photosynthesis. For example, mutants in the single-copy *SPPA1* gene in *Arabidopsis thaliana* maintain a higher level of the quantum efficiency of Photosystem II [14]. The photosynthetic ability of parasitic plants ranges from reduced levels to a complete lack of the ability to photosynthesize [2]. Most *Cuscuta* species do not have chlorophyll and thus, cannot photosynthesize [15]. However, some *Cuscuta* species (*C. pentagona* and *C. reflexa*) have chloroplasts with photosystems and some chlorophyll [15–18]. A recent study showed that highly divergent plastid chromosomes exist in non-photosynthetic parasitic plants [19–22]. The size of plastid genome in *Cuscuta* species is related to their photosynthetic capacity. Photosynthetic species have more plastomes than non-photosynthetic species [23]. In addition, gene loss is significantly correlated with species in different subgenera or Sects.  [24, 25]. However, Revill et al. identified the loss of photosynthesis and alterations in the structure of the cp genome of 15 *Cuscuta* species using the DNA dot analysis method but did not find a correlation with the phylogenetic position [9]. Therefore, more studies are needed to assess the variation of the cp genome in *Cuscuta* species and their relationship with subgenera or sections, thus, providing important information on the evolution of *Cuscuta* species.

Both *C. epithymum* and *C. europaea* had been proposed to belong to subgenus *Cuscuta* [12], however, no complete cp genome was available until now. Both *C. chinensis* and *C. japonica* collected in Korea were identified to belong to subgenus *Grammica* and *Monogynella*, respectively, based on the complete cp genome sequences [26]. *C. gronovii* was identified to belong to subgenera *Grammica* [26] based on the complete cp genome sequences [27]. In this study, five *Cuscuta* species, including *C. epithymum*, *C. europaea*, *C. gronovii*, *C. chinensis* and *C. japonica*, were sequenced, and their cp genomes were assembled. We then compared the whole cp genome of 23 *Cuscuta* species to determine the following: (1) the novel cp genomes of both *C. epithymum* and *C. europaea*; (2) the phylogenetic relationship based on the whole cp genomes of the 23 *Cuscuta* species and the division of four subgenus; (3) the structural variation of the cp genomes among the 23 *Cuscuta* species, including *C. chinensis*, *C. japonica*, and *C. gronovii* collected in China; and (4) the loss of genes in the cp genome of the 23 *Cuscuta* species and its correlation with phylogenetic positions and photosynthetic ability. This study uncovered the phylogenetic relationships and variations in the cp genomes of *Cuscuta* species.

## Results

### Cp genome features of five *Cuscuta* species

**Fig. 1:**
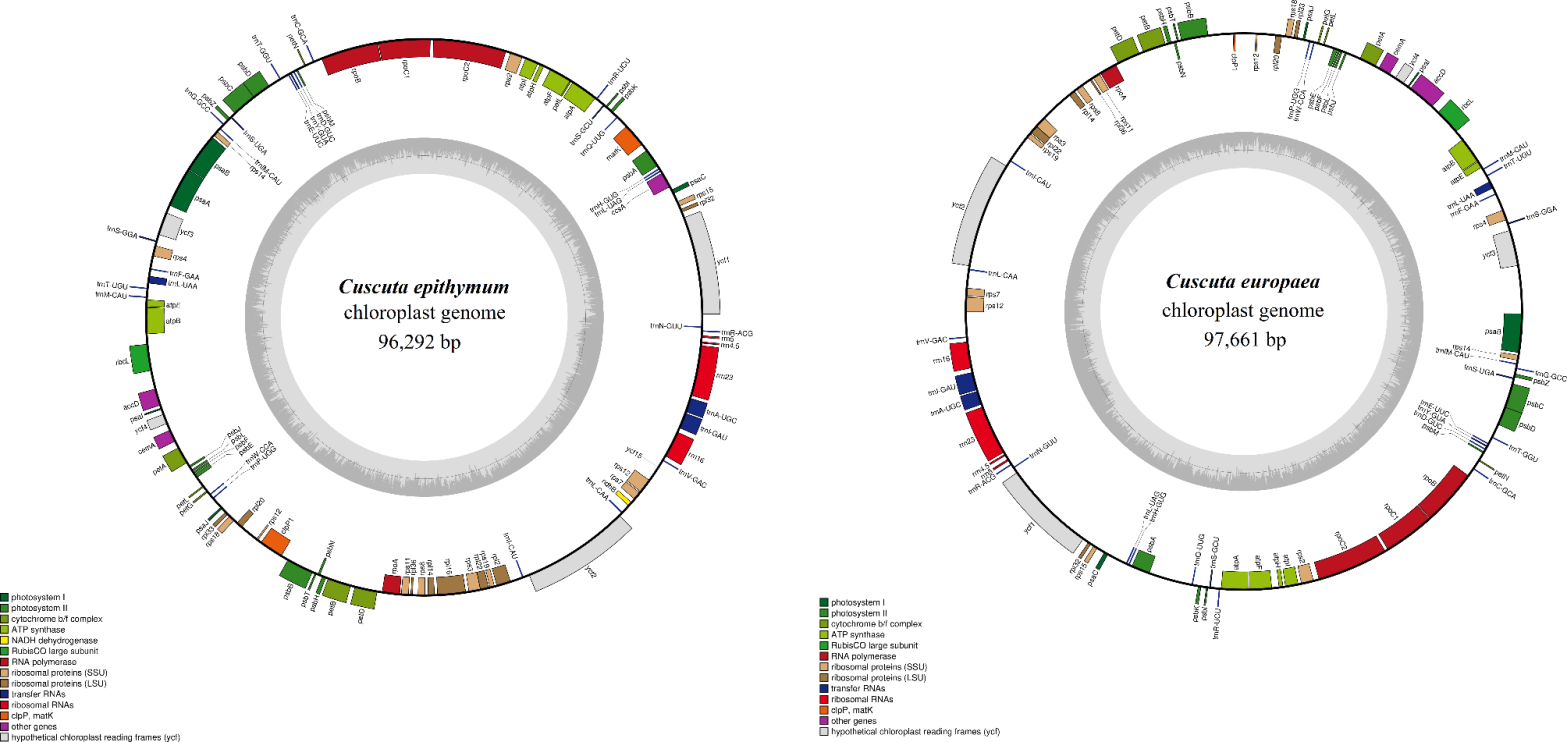
Genetic map of the chloroplast genomes of *Cuscuta epithymum* and *C. europaea*. The transcriptional direction of genes in the outside circle is counter-clockwise, while those in the inside circle are clockwise. The outer circle shows the genes at each locus. The inner circle also shows the GC content graph of the genome, where the dark and light gray lines indicate the GC and AT contents, respectively, at each locus

The cp genomes of five *Cuscuta* species were sequenced and the raw data ranged from 11,769,885 (*C. gronovii*) to 273, 318,504 (*C. japonica*), while the clean data ranged from 11,646,979 (*C. gronovii*) to 273,081,185 (*C. japonica*) (Table 1). Assembled by NOVOPlasty (version 3.7.2), the length of the cp genomes of five *Cuscuta* species ranged from 86,745 bp (*C. gronovii*) to 121,031 bp (*C. japonica*) (Table 2). Among them, the cp genomes of *C. chinensis*, *C. japonica* and *C. gronovii* were 99.87%, 100%, and 99.86% similar with those deposited in the NCBI database (Table 2). The cp genomes of *C. epithymum* and *C. europaea* were novel. The cp genome sequences of *C. epithymum* and *C. europaea* were similar with that of *C. approximata* with a similarity of 97.96% and 95.45%, respectively. The complete cp genome sequences of *C. epithymum* and *C. europaea* were 96,292 and 97,661 bp long, respectively, and lacked an inverted repeat (IR) region (Fig. 1). The LSC regions were 69,214 bp and 77,514 bp long in *C. epithymum* and *C. europaea*, respectively, and those of the SSC region were 2,908 bp and 1,624 bp long, respectively. The IR regions (IRa and IRb) were 24,170 bp and 18,523 bp in *C. epithymum* and *C. europaea*, respectively. The GC content of *C. epithymum* and *C. europaea* was 37.7% and 37.6%, respectively (Table 2). The gene content and gene order differed substantially between the two *Cuscuta* Cp genomes. The cp genome of *C. epithymum* harbored 99 unique genes, including 66 protein-coding genes, four rRNA genes and 27 tRNA genes, whereas that of *C. europaea* contained 91 unique genes, including 60 protein-coding genes, four rRNA genes and 27 tRNA genes (Table 2). Genetic map of the cp genomes of *C. japonica, C. gronovii and C. chinensis* were in the supplement (Figure S1, S2 and S3).

**Table 1:** The data of NGS sequencing of the five *Cuscuta* species

*Cuscuta* species	Raw reads	Clean reads	Coverage
*C. japonica*	273,318,504	273,081,185	2256×
*C. gronovii*	11,769,885	11,646,979	134×
*C. chinensis*	158,489,958	158,331,468	1821×
*C. epithymum*	53,143,010	52,404,322	544×
*C. europaea*	46,230,054	45,467,258	465×

**Table 2:** Characteristics of the chloroplast genomes of 23 *Cuscuta* species and the outgroup

Species	Genbank accession No.	Total length(bp)	LSC length	SSC length	IR length	genes	tRNA	rRNA	CDS	CG%	reference
*C. exaltata*	EU189132	125,373	83,320	8571	33,482	124	42	8	69	38.1	McNeal et al. (2007)
*C. reflexa*	AM711640	121,521	79,468	8571	33,482	112	35	8	68	38.2	Funk et al. (2007)
*C. japonica*	MH780080	121,037	79,517	8412	33,108	107	32	8	67	38.3	Unpublished
*C. japonica*	OL752640	121,031	79,505	8388	33,138	107	32	8	67	38.3	this study
*C. nitida*	NC052869	113,762	69,480	8012	36,270	106	30	8	68	37.5	Banerjee and Stefanović (2020)
*C.africana*	NC052870	105,066	60,830	7580	36,656	104	30	8	62	37.5	Banerjee and Stefanović (2020)
*C. approximata*	NC052871	98,380	91,011	7369	N/A	96	27	4	64	35.0	Banerjee and Stefanović (2020)
*C. pedicellata*	MN464181	97,091	89,375	7716	N/A	96	27	4	64	35.4	Banerjee and Stefanović (2020)
*C. epithymum*	OP620588	96,292	69,214	2908	24,170	97	27	4	66	35.1	This study
*C. europaea*	OP620589	97,661	77,514	1624	18,523	91	27	4	60	35.2	This study
*C. chinensis*	MH780079	86,927	50,572	7121	29,234	96	26	8	62	37.6	Unpublished
*C. chinensis*	OL752638	86,910	50,547	7129	29,234	98	28	8	62	37.6	this study
*C. gronovii*	AM711639	86,744	50,973	7063	28,708	98	28	8	61	37.7	Funk et al. (2007)
*C. gronovii*	OL752639	86,745	50,974	7063	28,708	98	28	8	61	37.7	this study
*C. campestris*	NC052920	86,727	50,956	7063	28,708	96	28	8	60	37.7	Unpublished
*C. costaricensis*	MK881072	86,691	50,149	7354	29,188	96	28	8	60	37.1	Banerjee and Stefanović (2019)
*C. pentagona*	MH121054	86,380	50,958	7022	28,400	97	28	8	61	37.9	Park et al. (2018)
*C. obtusiflora*	EU189133	85,286	50,207	6817	28,262	98	29	8	61	37.8	McNeal et al. (2007)
*C. australis*	NC045885	85,263	50,384	6727	28,152	97	28	8	61	37.8	Wang et al. (2020)
*C. chapalana*	MK887214	84,607	50,250	8223	26,134	96	27	8	61	37.6	Banerjee and Stefanović (2019)
*C. mexicana*	MK887213	83,526	50,154	6894	26,478	92	25	8	57	37.5	Banerjee and Stefanović (2019)
*C. bonafortunae*	MK887215	82,346	49,128	7920	25,298	95	27	8	59	37.3	Banerjee and Stefanović (2019)
*C. carnosa*	MK887212	81,577	48,410	8729	24,438	91	23	8	58	37.8	Banerjee and Stefanović (2019)
*C. strobilacea*	MK867795	63,787	30,720	6863	26,204	74	27	8	33	37.1	Banerjee and Stefanović (2019)
*C. boldinghii*	MK881074	62,375	30,090	6701	25,584	70	27	8	31	36.8	Banerjee and Stefanović (2019)
*C. erosa*	MK881073	60,959	29,596	6275	25,088	71	27	8	33	36.9	Banerjee and Stefanović (2019)
*I. purpurea*	EU118126	162,046	88,172	12,110	61,764	140	45	8	87	37.5	McNeal et al. (2007)

*Cuscuta* species have genomic sequence lengths that range from 60,905 bp to 125,373 bp. *Cuscuta* cp genomes have 31–69 protein-coding genes, 23–42 transfer RNAs (tRNAs), and 4–8 ribosomal RNAs (rRNAs) (Table 2). The most diminished cp genome, that of *C. erosa* (60,959 bp long), has 33 protein-coding genes, 27 tRNAs, and eight rRNAs and was reduced by 62% compared with the chloroplast. The genome of *Ipomoea purpurea*, a member of the Convolvulaceae family, was used as the reference genome. The cp genome of *C. exaltata* (125,373 bp long) has 69 protein-coding genes, 42 tRNAs, and eight rRNAs with a reduction in its composition of 22% that demonstrated a significant variation in the genome length and gene composition in the *Cuscuta* chloroplast. The cp genomes of *C. exaltata*, *C. reflexa*, and *C. japonica* were larger than the genomes of remaining *Cuscuta* species (24-25% sequence reduction compared with the genome of *I. purpurea*) (Table 2).

### Phylogenetic analysis

**Fig. 2:**
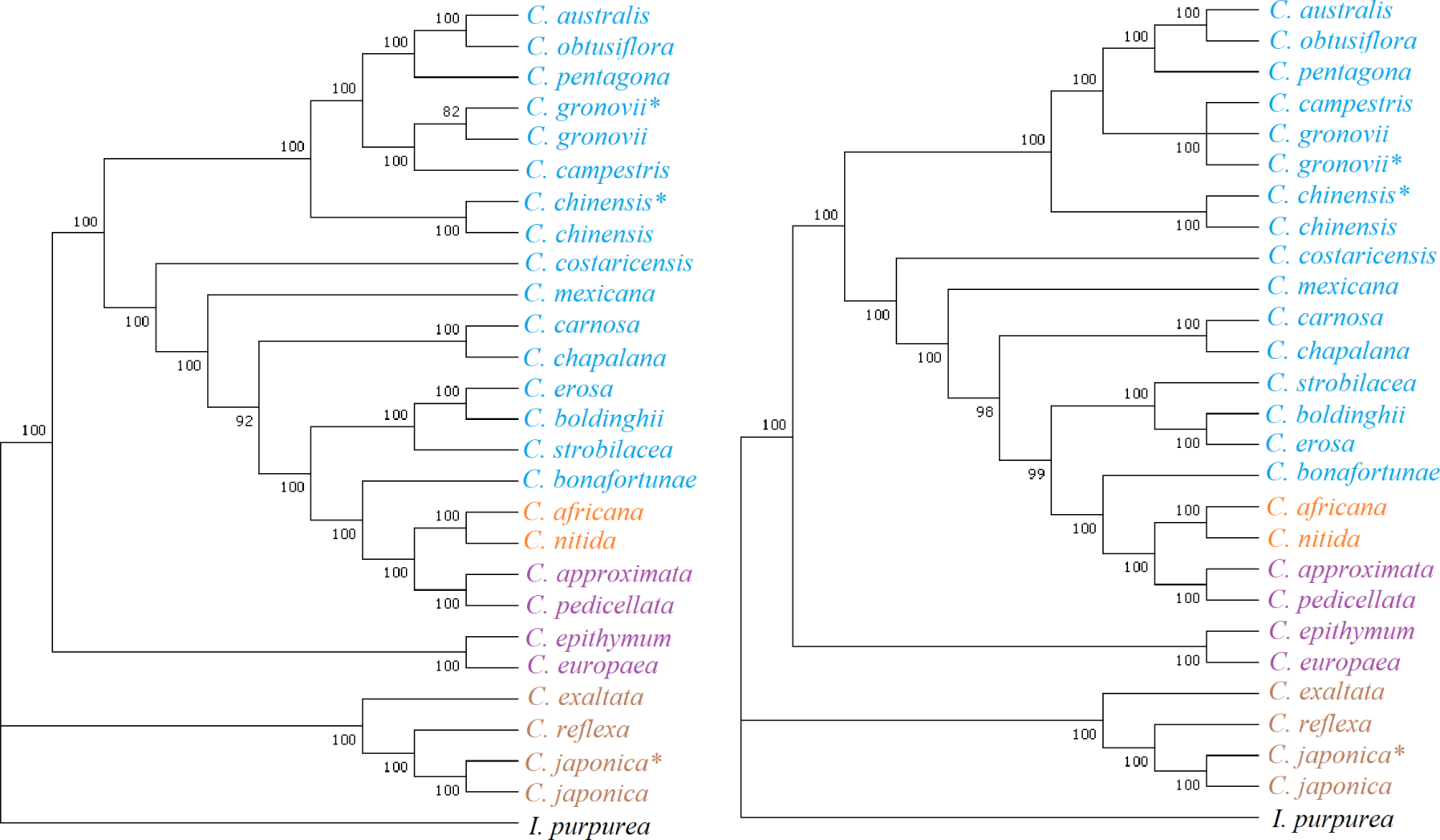
The phylogenetic trees of 23 *Cuscuta* species and *Ipomoea purpurea* as determined from different data based on protein-coding sequences (left) and the complete chloroplast genome sequences (right). The support values are shown for nodes as maximum likelihood bootstrap (approach branches). The species with the same color belong to the same subgenus. Brown, purple, orange and blue represent the subgenera *Monogynella*, *Cuscuta*, *Pachystigma*, and *Grammica*, respectively. * indicates that the chloroplast genomes of these *Cuscuta* species were sequenced in this study

The GTR + G + I model was selected as the best-fit substitution model using MEGA 7. Herein, phylogenetic trees based on protein-coding sequences and complete cp genome sequences produced similar topologies (Fig. 2). The 23* Cuscuta* species clustered into four subgenera, *Monogynella*, *Cuscuta*, *Pachystigma*, and *Grammica*. The subgenus *Monogynella* contains *C. exaltata*, *C. reflexa*, and *C. japonica*. The subgenus *Cuscuta* includes *C. epithymum*, *C. europaea*, *C. approximata* and *C. pedicellata*. The subgenus *Pachystigma* includes *C. nitida* and *C. africana*. The remaining species form the subgenus *Grammica*.

### Simple sequence repeats (SSRs) analysis

A total of 471 SSRs were detected in the 23 *Cuscuta* species (Table 3). Among them, more than 80% were mononucleotide SSRs and belonged to the A or T types. Only one SSR in *C. approximata* and one SSR in *C. europaea* were polynucleotide repeats belonging to the c type.

**Table 3:** Chloroplast SSRs in 23 *Cuscuta* species

	Total	c	p1	Single nucleotide repeats							
				(A)10	(A)11	(A)12	(A)others	(T)10	(T)11	(T)12	(T)others
*C. exaltata*	31	0	2	9	3	2	0	6	4	3	2
*C. reflexa*	36	0	4	9	3	3	2	7	3	4	1
*C. japonica*	21	0	0	4	3	0	0	7	6	1	0
*C. japonica* (this study)	22	0	0	4	1	1	1	4	4	2	5
* C. nitida*	46	0	2	13	5	2	6	7	2	1	8
*C. africana*	37	0	2	12	4	1	2	9	2	2	3
*C. approximata*	52	1	1	11	6	1	5	10	4	3	10
*C. pedicellata*	52	0	5	14	6	4	9	7	4	1	2
*C. epiyhymum* (this study)	43	0	2	10	5	5	0	12	6	3	0
* C. europaea* (this study)	34	1	1	7	1	2	3	13	3	2	1
* C. chinensis*	20	0	1	0	3	0	0	2	6	2	6
*C. chinensis* (this study)	20	0	2	0	3	0	0	1	7	1	6
* C. gronovii*	22	0	0	3	1	1	1	4	4	2	6
*C. gronovii* (this study)	22	0	0	3	1	1	1	4	4	2	6
* C. campestris*	15	0	0	3	1	1	1	4	4	2	6
*C. costaricensis*	9	0	0	0	2	0	0	3	0	1	3
*C. pentagona*	22	0	0	4	2	1	1	6	4	3	1
*C. obtusiflora*	28	0	0	3	3	4	4	7	2	2	3
*C. australis*	24	0	0	6	1	4	2	5	4	1	1
*C. chapalana*	27	0	4	1	4	0	2	5	4	2	5
*C. mexicana*	37	0	1	2	3	2	4	7	4	5	9
*C. bonafortunae*	40	0	1	4	3	0	11	6	7	2	6
*C. carnosa*	29	0	0	5	1	0	0	8	6	1	8
*C. strobilacea*	16	0	0	3	2	1	0	5	3	0	2
*C. boldinghii*	29	0	2	4	4	0	6	3	4	2	4
*C. erosa*	21	0	1	1	3	1	5	4	2	1	3

p1: Dinucleotide Repeats; c: indicates Polynucleotide repeat. A: Adenine; T: Thymine

### Sequence inversions

**Fig. 3:**
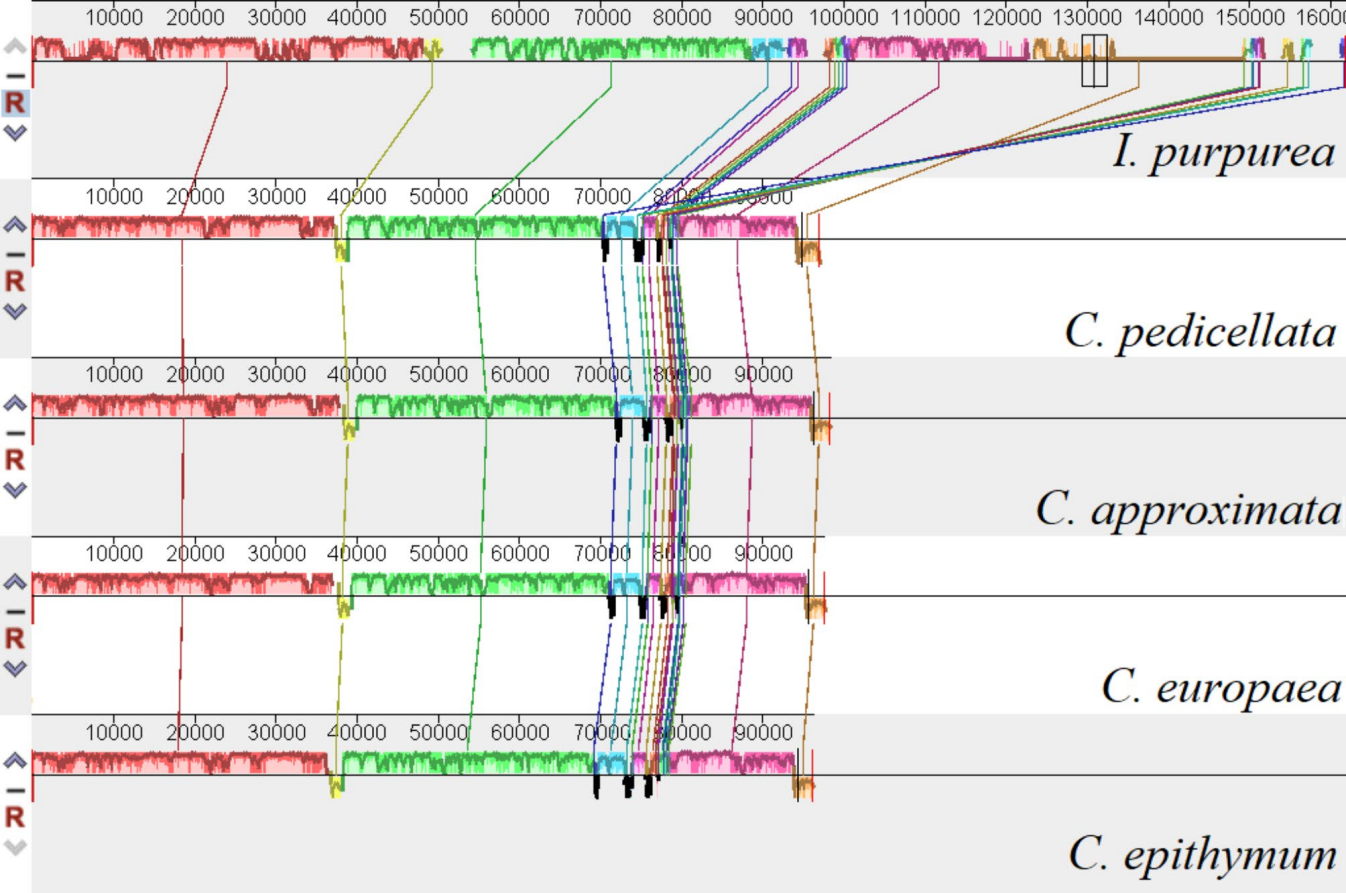
Structural variation of chloroplast genomes among *C. epithymum*, *C. europaea*, *C. approximata*, and *C. pedicellata* belonging to subgenus *Cuscuta* compared with genome of *Ipomoea purpurea*. The yellow region represents inversion A, and the brown region represents inversion B. The four black regions represent the four inversions. The colored regions (red, green, blue and pink) do not contain gene inversions

Compared with the genome of *I. purpurea*, the structural changes in the sequences among *C. epithymum*, *C. europaea*, *C. approximata*, and *C. pedicellata* belonging to subgenus *Cuscuta* were shown in Fig. 3. Two sequence inversions were detected in the subgenera of *Cuscuta*. One inversion included *trnL-UAA*, *trnT-UGU* and *trnF-GAA* (inversion A), while the other included *ccsA*, *psaC* and *rps15* (inversion B). There were four inversions (black region) that did not contain any genes (Fig. 3).

### IR expansion and contraction

**Fig. 4:**
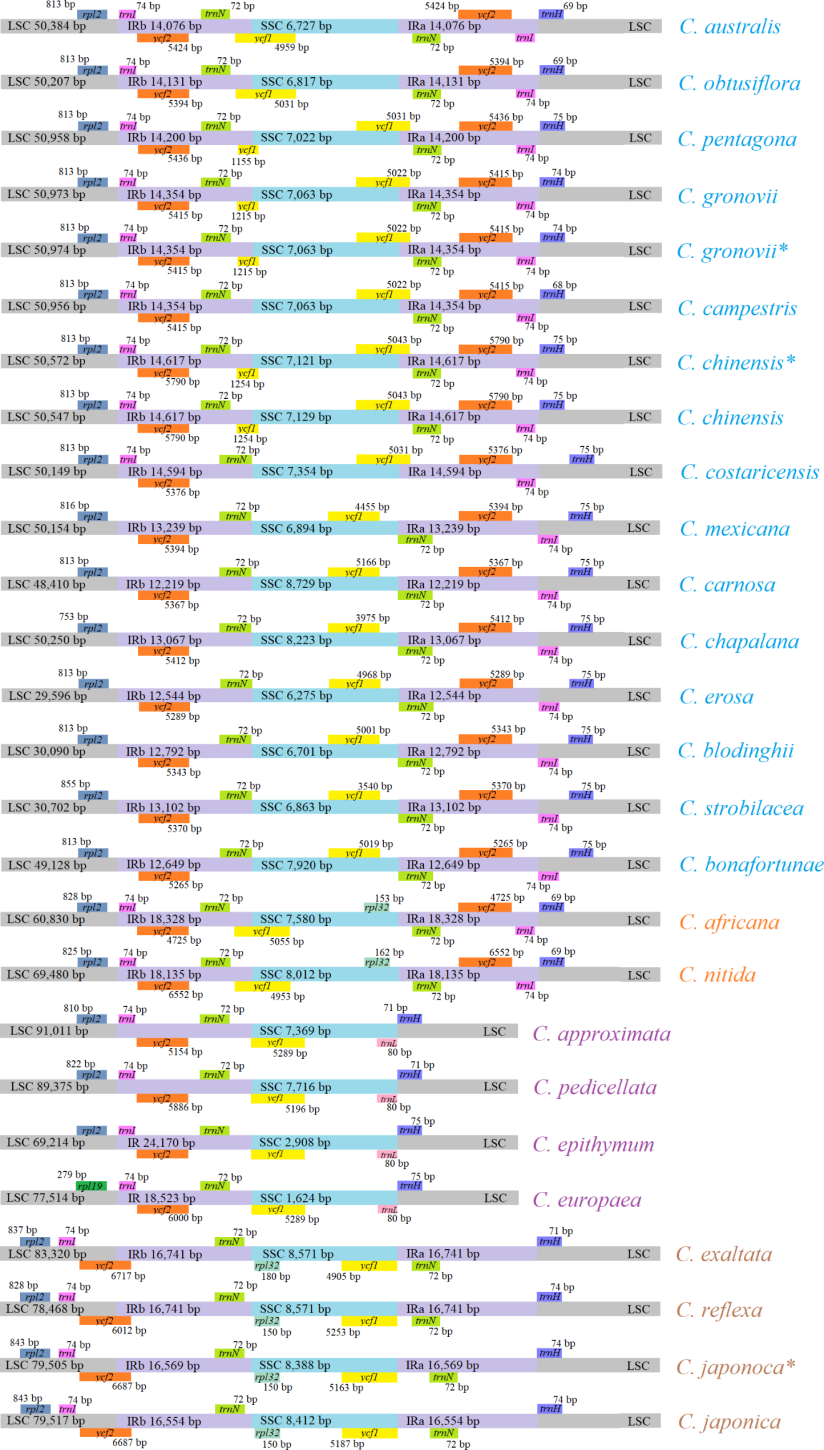
The IR borders in chloroplast genomes of 23 *Cuscuta* species. The gray, purple, and light blue blocks represent the LSC region, IR region and SSC region, respectively. Blocks with different colors represent different genes. IR, inverted repeat; LSC, large single-copy; SSC, small single-copy

Expansion and contraction at the IR region boundaries are common and influence the variation in the sizes of cp genomes. A detailed comparison between the IR-SSC and IR-LSC borders of genomes among the 23 intact four-part structures (IR-SSC-IR-LSC) of the *Cuscuta* chloroplasts is shown in Fig. 4. Similar to the sequence inversions, the IR borders were highly conserved within the *Cuscuta* subgenus. The *ycf2* gene crossed the LSC/IRb region of species in the subgenus *Monogynella*, including *C. reflexa*, *C. japonica*, and *C. exaltata*. The length of extension of the *ycf2* gene into the LSC region was based on the genome (*C. reflexa*, 3,519 bp; *C. japonica*, 4,228 bp; and *C. exaltata*, 4,227 bp). The IRb/SSC junction of the subgenus *Pachystigma* was located in the *ycf1* gene and extended into the IRb regions (1,534 bp in *C. africana* and 1,062 bp in *C. nitida*). The IR boundaries varied significantly more in subgenus *Grammica* (four *ycf1* distribution patterns that crossed the IR boundaries) compared with the subgenera *Monogynella* and *Pachystigma*. The first one was located inside the SSC regions (*C. erosa*, *C. boldinghii*, *C. strobilacea*, *C. carnosa*, *C. bonafortunae*, *C. mexicana*, and *C. chapalana*). The second extended by ~ 1,000 bp from the SSC region to the IRa region (*C. gronovii*, *C. campestris*, and *C. costaricensis*); the third one extended by less than 1,000 bp from the SSC region to IRb region (*C. obtusiflora* and *C. australis*), and the last one crossed both the SSC/IRa (extended by ~ 1,000 bp to IRa) and the SSC/IRb regions (extended by 258 and 956 bp to the IRb, respectively) (*C. chinensis* and *C. pentagona*). In addition, in contrast to the other *Cuscuta* species, the cp genomes of *C. epithymum*, *C. europaea*, *C. approximata* and *C. pedicellata* lacked an IR.

### Patterns of reduction of the *Cuscuta* cp. genome

The 23 *Cuscuta* species had a significantly reduced genome size and gene content (Tables 2 and 4). Notably, the entire NAD(P)H dehydrogenase complex gene (*ndh*) family related to land adaptation and photosynthesis was lost in all 23 *Cuscuta* species. The photosystem genes (*ycf15* and *ycf1*) and ribosomal protein-coding genes (*rpl23*, *rps15*, and *rps16*) were lost in the 23 *Cuscuta* cp genomes. In addition, *matK* that encodes maturase and the photosynthesis-related gene *psaI* were lost in all the species in subgenus *Grammica*. The photosystem gene *ycf2* was lost in the three subgenera *Cuscuta*, *Pachystigma*, and *Monogynella*. The lost genes were relatively conserved inside subgenus *Cuscuta* (Fig. 3). However, all the ATP synthase genes (*atp*) were present in all 23 *Cuscuta* species, even in the smallest cp genome *C. erosa* (except for *atpF* in *C. boldinghii*). *TrnA-UGC*, *trnG-UCC, trnI-GAU*, *trnK-UUU*, *trnL-UAA*, *trnV-UAC* were lost in all 23 species. Two copies of *trnR-ACG* were only lost in all the species of subgenus *Grammica.*

**Table 4:** Loss of chloroplast protein coding genes and transfer RNA genes across the *Cuscuta* spp

Species	Subgenera	Lost genes			
		Photosynthesis related genes	Transcription and translation related genes	Other genes	tRNA genes
*C. erosa*	*Grammica*	*ndh*, *psaI*, *psaA-C*, *psbA-F*, *psbH-L*, *psbN/T/Z*, *petA/B/D/G/L/N*, *rbcL*	*rpl23*, *rps15*, *rps16*, *rpo*	*ycf1*, *ycf3*, *ycf4*, *matK*, *ccsA*, *cemA*, ycf15**	trnA-UGC****, trnG-UCC**, trnI-CAU, trnI-GAU**, trnI-GAU**, trnK-UUU**, trnL-UAA, trnR-ACG, trnR-ACG, trnV-UAC**
*C. boldinghii*		*ndh*, *psaI*, *psaA-C*, *psbA-F*, *psbH-L*, *psbT*, *psbZ*, *petA/B/D/G/L/N*, *rbcL*, *atpF*	*rpl23*, *rpl36*, *rpl32*, *rps14*, *rps15*, *rps16*, *rpo*	*ycf1*, *ycf3*, *ycf4*, *matK*, *ccsA*, ycf15**	trnA-UGC****, trnG-GCC, trnG-UCC, trnI-CAU, trnI-GAU**, trnI-GAU**, trnK-UUU**, trnL-UAG, trnR-ACG**, trnV-UAC**
*C. strobilacea*		*ndh*, *psaI*, *psaA-C*, *psaJ*, *psbA-F*, *psbT*, *psbZ*, *psbH/J/K/L*, *petA/B/D/G/L/N*, *rbcL*	*rpl23*, *rpl32*, *rpl36*, *rps15*, *rps16*, *rpo*	*ycf1*, *ycf3*, *ycf4*, *matK*, *ccsA*, ycf15**	trnA-UGC****, trnG-UCC**, trnI-CAU, trnI-GAU****, trnK-UUU**, trnL-UAA, trnR-ACG**, trnV-UAC**
*C. carnosa*		*ndh*, *psaI*, *petN*, *psbM*	*rpl23*, *rpl32*, *rps15*, *rps16*, *rpo*	*ycf1*, *matK*, *ccsA*, ycf15**	trnA-UGC****, trnC-GCA, trnD-GUC, trnE-UUC, trnG-UCC**, trnI-CAU, trnI-GAU****, trnK-UUU**, trnL-UAA, trnR-ACG**, trnV-GAC, trnV-UAC, trnY-GUA
*C. bonafortunae*		*ndh*, *psaI*, *psbL*	*rpl23*, *rpl32*, *rpl36*, *rps15*, *rps16*, *rpo*	*ycf1*, *matK*, ycf15**	trnA-UGC****, trnG-UCC**, trnI-CAU, trnI-GAU****, trnK-UUU**, trnL-UAA, trnR-ACG**, trnV-UAC**
*C. mexicana*		*ndh*, *psaI*, *rbcL*, *psbI*, *psbK*	*rpl23*, *rpl32*, *rps15*, *rps16*, *rpo*	*ycf1*, *matK*, *cemA*, ycf15**	trnA-UGC****, trnG-UCC**, trnI-CAU, trnI-GAU****, trnK-UUU**, trnL-UAA, trnR-ACG**, trnR-UCU, trnS-GCU, trnV-UAC**
*C. chapalana*		*ndh*, *psaI*	*rpl23*, *rpl32*, *rps15*, *rps16*, *rpo*	*ycf1*, *matK*, ycf15**	trnA-UGC****, trnG-UCC**, trnI-CAU, trnI-GAU****, trnK-UUU**, trnL-UAA, trnR-ACG**, trnV-UAC**
*C. costaricensis*		*ndh*, *psaI*, *atpI*	*rpl23*, *rpl32*, *rps15*, *rps16*, *rpo*	*ycf1*, *matK*, ycf15**	trnA-UGC****, trnG-UCC**, trnI-GAU****, trnK-UUU**, trnL-UAA, trnR-ACG**, trnV-UAC**
*C. australis*		*ndh*, *psaI*	*rpl23*, *rpl32*, *rps15*, *rps16*, *rpo*	*ycf1*, *matK*, ycf15**	trnA-UGC****, trnG-UCC**, trnI-GAU****, trnK-UUU**, trnL-UAA, trnR-ACG**, trnV-UAC**
*C. obtusiflora*		*ndh*, *psaI*	*rpl23*, *rpl32*, *rps15*, *rps16*, *rpo*	*ycf1*, *matK*, ycf15**	trnA-UGC****, trnG-UCC**, trnI-GAU****, trnK-UUU**, trnR-ACG**, trnV-UAC**
*C. campestris*		*ndh*, *psaI*	*rpl23*, *rpl32*, *rps15*, *rps16*, *rps12*, *rpo*	*ycf1*, *matK*, ycf15**	trnA-UGC****, trnG-UCC**, trnI-GAU****, trnK-UUU**, trnL-UAA, trnR-ACG**, trnV-UAC**
*C. pentagona*		*ndh*, *psaI*, *atpE*	*rpl23*, *rpl32*, *rps15*, *rps16*, *rpo*	*matK*, ycf15**	trnA-UGC****, trnG-UCC**, trnI-GAU****, trnK-UUU**, trnL-UAA, trnR-ACG**, trnV-UAC**
*C. gronovii*		*ndh*, *psaI*	*rpl23*, *rpl32*, *rps15*, *rps16*, *rpo*	*ycf1*, *matK*, ycf15**	trnA-UGC****, trnG-UCC**, trnI-GAU****, trnK-UUU**, trnL-UAA, trnR-ACG**, trnV-UAC**
*C. gronovii* (this study)		*ndh*, *psaI*	*rpl23*, *rpl32*, *rps15*, *rps16*, *rpo*	*ycf1*, *matK*, ycf15**	trnA-UGC****, trnG-UCC**, trnI-GAU****, trnK-UUU**, trnL-UAA, trnR-ACG**, trnV-UAC**
*C. chinensis*		*ndh*, *psaI*	*rpl23*, *rpl32*, *rps15*, *rps16*, *rpo*	*matK*, ycf15**	trnA-UGC****, trnG-UCC**, trnI-GAU****, trnK-UUU**, trnL-UAA**, trnR-ACG**, trnT-GGU, trnV-UAC**
*C. chinensis* (this study)		*ndh*, *psaI*	*rpl23*, *rpl32*, *rps15*, *rps16*, *rpo*	*matK*, ycf15**	trnA-UGC****, trnG-UCC**, trnI-GAU****, trnK-UUU**, trnL-UAA**, trnR-ACG**, trnT-GGU, trnV-UAC**
*C. epithymum* (this study)	*Cuscuta*	*ndh*	*rpl23, rps7, rps12, rps15*	*ycf1*, *ycf2*, ycf15**	trnA-UGC^***^, trnG-UCC^**^, trnI-GAU^***^, trnK-UUU^**^, trnL-CAA, trnL-CAA, trnV-GUU, trnR-ACG, trnV-GAC^**^
*C. europaea* (this study)		*ndh, psaA*	*rpl16, rpl2, rpl23, rps12, rps15, rps4, rps7*	*ycf1*, *ycf2*, ycf15**, *matK*, *ccsA*	trnA-UGC^***^, trnG-UCC^**^, trnI-CAU, trnI-GAU^**^, trnK-UUU^**^, *trnL-CAA, trnL-UAA, trnN-GUU, trnR-ACG, trnV-GAC, trnV-UAC*
*C. pedicellata*		*ndh*	*rpl23*, *rps15*, *rps16*, *rps7*, *rps12*, *rpoC2*	*ycf1*, *ycf2*, ycf15**	trnA-UGC***, trnG-UCC**, trnI-CAU, trnI-GAU***, trnK-UUU**, trnL-CAA, trnL-UAA, trnN-GUU, trnR-ACG, trnV-GAC, trnV-UAC**
*C. approximata*		*ndh*	*rpl23*, *rps15*, *rps16*, *rps7*, *rps12*, *rpoC2*	*ycf1*, *ycf2*, ycf15**	trnA-UGC***, trnG-UCC**, trnI-CAU, trnI-GAU**, trnK-UUU**, trnL-CAA, trnL-UAA, trnN-GUU, trnR-ACG, trnV-GAC, trnV-UAC**
*C. africana*	*Pachystigma*	*ndh*, *psbZ*	*rpl23*, *rps15*, *rps16*, *rpo*	*ycf1*, ycf15**, *clpP*	trnA-UGC****, trnG-UCC**, trnI-GAU****, trnK-UUU**, trnL-UAA, trnV-UAC**
*C. nitida*		*ndh*	*rpl23*, *rps15*, *rps16*	*ycf1*, ycf15**	trnA-UGC****, trnG-UCC**, trnI-GAU****, trnK-UUU**, trnL-UAA, trnV-UAC**
*C. japonica*	*Monogynella*	*ndh*	*rpl23*, *rps15*, *rps16*	*ycf1*, *ycf2*, ycf15**	trnA-UGC**, trnG-UCC**, trnI-CAU, trnI-GAU**, trnK-UUU**, trnL-UAA, trnV-UAC**
*C. japonica* (this study)		*ndh*	*rpl23*, *rps15*, *rps16*	*ycf1*, *ycf2*, ycf15**	trnA-UGC**, trnG-UCC**, trnI-CAU, trnI-GAU**, trnK-UUU**, trnL-UAA, trnV-UAC**
*C. reflexa*		*ndh*	*rpl23*, *rps15*, *rps16*	*ycf1*, *ycf2*, ycf15**	trnA-UGC**, trnG-UCC, trnI-CAU, trnI-GAU**, trnK-UUU**, trnL-UAA, trnV-GAC**
*C. exaltata*		*ndh*	*rpl23*, *rps15*, *rps16*	*ycf1*, *ycf2*, ycf15**	trnA-UGC**, trnG-UCC, trnI-CAU, trnI-GAU**, trnK-UUU**, trnL-UAA, trnV-UAC

**gene loss two copies. *** gene loss three copies. **** gene loss four copies

## Discussion

### Molecular phylogeny of 23 *Cuscuta* species

Phylogenetic analysis of specific *Cuscuta* species is necessary to clarify the phylogenetic location of each species. To date, 23 plastomes have been identified [4, 10, 12, 25, 27, 28], including *C. europaea* and *C. epithymum* obtained in this study. Here, we found that *C. europaea* was closely related to *C. epithymum* based on protein coding genes and whole cp genome. This result was consistent with the findings of Neumann [29]. We also found that *C. australis* is closely related to *C. pentagona*, which is consistent with the findings of previous research [4], and not closely related to *C. epithymum*, which was mentioned by Revill et al. [9]. These comparative genomic analyses provide new insights into understanding the phylogeny of *Cuscuta* species. However, it is necessary to increase the sample size of *Cuscuta* species and use data based on nuclear genome sequencing to strengthen the understanding of their phylogenetic relationships.

### Division of four subgenera

The division of four subgenera (*Monogynella*, *Cuscuta*, *Pachystigma*, and *Grammica*) was always controversial. Banerjee and Stefanović classified six *Cuscuta* species into four subgenera using the whole cp genome sequencing method and found it was consistent with the morphological and DNA sequences-based phylogenetic method, however, the number of studied species was limited [12]. In this study, we classified 23 *Cuscuta* species into four subgenera based on the complete cp genome sequences and found it was not consistent with the phylogenetic relationship.

Neumann (2020) divided *C. approximate* and *C. pedicellata* into the subgenus *Cuscuta* [29] and both *C. epithymum* and *C. europaea* were identified to belong to subgenus *Cuscuta* by using sequences of the nuclear ribosomal internal transcribed spacer and plastid *rps2*, *rbcL* and *matK* [10]. The cp genomes of *C. europaea*, *C. epithymun, C. approximate* and *C. pedicellata* had the same pattern of gene inversion and both lacked one IR region (Figs. 3 and 4). Therefore, we agreed that *C. europaea* and *C. epithymum* belonged to subgenus *Cuscuta*. However, in this study, *C. europaea* and *C. epithymum* grouped together, while *C. approximate* and *C. pedicellata* formed another group, however, the two clades were not sister to each other. McNeal et al. found that subgenus *Cuscuta* is unequivocally paraphyletic with subgenus *Grammica* and subgenus *Pachystigma* nested within it [10]. Thus, we hypothesized that *C. europaea* and *C. epithymum* might be classified into another subgenus and played special role in the morphological and plastid genome evolution. More studies including more numbers of *Cuscuta* species are needed in the future.

The subgenus *Monogynella*, containing of *C. exaltata*, *C. reflexa*, and *C. japonica*, was monophyletic and was the most basal clade with fewer speciation events. The *ycf2* gene crossed the LSC/IRb region of species in *C. reflexa*, *C. japonica*, and *C. exaltata*. The results were consistent with the findings of McNeal et al. [10] and Park et al. [26].

In this study, the molecular phylogeny showed *C. nitida* and *C. africana* were closed to *C. approximate* and *C. pedicellate* (Fig. 2), however, the cp genome of *C. nitida* and *C. africana* didn’t lose one IR region (Fig. 4). Thus, we suggested that *C. nitida* and *C. africana* belonged to subgenus *Pachystigma*, which was consistent with the findings of Banerjee and Stefanovic [12].

Wang et al. [4] and Banerjee and Stefanovic [25] revealed that *C. bonafortuna* was closed to *C. strobilacea* and were belonged to subgenus *Grammica*. In our research, the cp genome of *C. bonafortuna* didn’t loss one IR region which was different from *C. approximate* and *C. pedicellata* (Fig. 4). The four subgenus of *Cuscuta* occurred different gene loss event (Table 4). *C. bonafortuna* lost *ndh*, *psaI*, *psbL*, *rpl23*, *rpl32*, *rps15*, *rps16*, *ycf1*, *matK*, *ycf15*, but *C. nitida* and *C. africana* both lost *rpl23, rps15, rps16, ycf1* and *ycf15*. The IR borders of *C. bonafortuna* is similar to *C. strobilacea* (Fig. 4). These results supported that *C. bonafortuna* belonged to subgenus *Grammica*. As mentioned by McNeal et al. [10], subgenera *Cuscuta, Grammica* and *Pachystigma* were not monophyletic, indicating that more evolution events related with the gene loss might happened among them, which might drive the changes of morphological and physiological traits of *Cuscuta* species. More studies are needed to elucidate the relationships among *Cuscuta* species based on taxonomic, morphological, physiological and molecular evidences.

### Variation in chloroplast gene structure

The uniparentally inherited SSRs in cp genomes are valuable molecular markers owing to their high degree of variations even within an individual species [30, 31]. Herein, most of the cp genomes of the 23 *Cuscuta* species had single nucleotide repeats of A and T, which are similar to those of other species [32, 33]. The validation of SSRs of cp genomes should be done before it can be used to identify species and in population genetics and evolutionary research of *Cuscuta* and its relatives.

Most cp genomes had a quadripartite structure, which consisted of two IR regions separated by one LSC and one SSC region (Fig. 2). The genomic structure, gene content, gene order, and base composition are highly conserved in the IR regions in most plant chloroplasts [34]. Herein, four *Cuscuta* species (*C. epithymum*, *C. europaea*, *C. approximata* and *C. pedicellata*) lacked an IR region (Table 2). The loss of IR could be found in the cp genomes of higher plants [35]. These results further confirmed the structural plasticity of the chloroplast. Owing to unequal recombination and replication slippage, the expansion and contraction of IR regions caused the structural variations in the IR boundaries (IRb) (Fig. 4).

Inversion events could be owing to the activity of tRNA [36] and high GC content [37]. The regions that flank two inversions (inversion A and inversion B) contained tRNA gene sequences (Fig. 3), indicating that tRNA recombination promotes inversions in the plastid genomes [36]. However, the GC content in inversion flanking sequences was not consistently higher than the average GC content in the cp genome. Therefore, the patterns of sequence variations at inversion boundaries are more consistent with tRNA activity and not intragenomic recombination between regions with a high content of GC [36, 37].

### Cp genome reduction and gene selection in *Cuscuta* species

*Cuscuta* is a parasitic angiosperm that exists as a hemiparasite or holoparasite [38]. Herein, the 23 *Cuscuta* species had a significantly reduced genome size and gene content (Tables 2 and 4), which is consistent with the findings of previous studies [10, 25, 39]. The lost genes were conserved in the *Cuscuta* subgenus and correlated with the species classification and phylogenetic relationships. Previous studies showed that *Cuscuta* species is a phenomenon of irreversibly reducing their genes, i.e., genes cannot be regained once they are lost [25, 40]. According to our results, *ndh*, *ycf1*, *ycf15*, *rpl23*, *rps15*, and *rps16* were lost in the species in all four subgenera, while the *matK* and *pasI* genes were lost in the subgenus *Grammica*. Therefore, it can be inferred that the *ndh*, *ycf15*, *ycf1*, *rpl23*, *rps15*, and *rps16* genes were lost before the *pasI*, *rpo*, and *matK* genes, considering that the genes could not be regained once they are lost.

The absence of *ndh* genes in angiosperms is primarily related to the loss of photosynthetic function in parasitic plants [41]. The *ndh* genes encode NDH dehydrogenase complexes, which are closely related to light and action [42]. The loss of NDH complex reduces the dependence of *Cuscuta* on photosynthesis, which is lower than that of green plants [43]. All the *Cuscuta* species lost *ndh* genes, indicating the loss of photosynthetic capacity during their evolution from autotrophy to heterotrophy, which is similar to the results of a previous study [25]. However, the functions of *ycf1* and *ycf15* genes are unclear. The loss of *rpl23*, *rps15*, and *rps16* genes related to ribosomal protein formation could enable the adaptation of *Cuscuta* to parasitic life. Herein, the types of gene reduction that were tracked included *psaI*, *rpo* and *matK* in the subgenus *Grammica*. *psaI* is related to the function of photosystem I (PS I). The *rpo* gene (*rpoA*, *rpoB*, *rpoC1*, and *rpoC2*) is crucial for the production of chloroplasts [44]. The loss of *rpo* gene can affect protein synthesis in the chloroplast, thus, affecting the development of chloroplasts and photosynthesis [45]. *MatK* is related to RNA processing [46]. The loss of *psaI*, *rpo*, and *matK* can decrease the ability of chloroplasts to photosynthesize and produce materials, which renders *Cuscuta* more parasitic. Herein, all the *Cuscuta* species that lost *ndh*, *psaI*, *rpo*, and *matK* (except for *C. erosa*, *C. boldinghii*, *C. strobilacea*) could be hemiparasitic. However, *C. erosa*, *C. boldinghii*, *C. strobilacea* lost several genes associated with photosynthesis, such as *psa*, *psb*, *pet*, *rbcL*, which indicates the transition from hemiparasitic to holoparasitic. These results indicate that the loss of genes related to photosynthesis is a continuous process [25]. The *ndh* gene was lost first (in all species of *Cuscuta*), followed by the *psaI*, *rpo* and *matK* genes (*C. africana* and across the subgenera *Grammica* and *Cuscuta*), and the substantial loss of *psa*, *psb*, *pet*, *rbcL*, and other genes (*C. erosa*, *C. boldinghii*, *C. strobilacea*). This study supports the model of plastid evolution proposed by Banerjee and Stefanović [25].

## Materials and methods

### Plant materials and cp. genome sequences

*C. chinensis* seeds were purchased in Aohanqisidaowanzi town, Chifeng City, China. *C. japonica* seeds were collected from a field in Sanmen County, China. *C. gronovii* seeds were collected from the field of Taizhou University, Taizhou City, China. After germination, the plants were identified by Professor Beifen Yang from Taizhou University based on their morphological traits according to a standard reference. Voucher herbarium specimens from the three species were deposited at Zhejiang Provincial Key Laboratory of Plant Evolutionary Ecology and Conservation in Taizhou University. *Cuscuta epithymum* from a wild population was collected in the region of Eleshnitsa, Pirin Mt., Bulgaria, while *Cuscuta europaea* was collected in Zlatni Mostove locality, Vitosha Mt., Bulgaria. Voucher herbarium specimens from the two species were deposited at Department of Biochemistry, Faculty of Biology, Sofia University. All the five *Cuscuts* species are parasitic weeds without regulations of ecological protection.

The whole cp genome sequences of 21 *Cuscuta* genes, including *C. exaltata* (EU189132), *C. reflexa* (AM711640), *C. japonica* (MH780080), *C. nitida* (NC052869), *C. africana* (NC052870), *C. approximata* (NC052871), *C. pedicellata* (MN464181), *C. chinensis* (MH780079), *C. gronovii* (AM711639), *C. campestris* (NC052920), *C. costaricensis* (MK881072), *C. pentagona* (MH121054), *C. obtusiflora* (EU189133), *C. australis* (NC045885), *C. chapalana* (MK887214), *C. mexicana* (MK887213), *C. bonafortunae* (MK887215), *C. carnosa* (MK887212), *C. strobilacea* (MK867795), *C. boldinghii* (MK881074), *C. erosa* (MK881073), and *I. purpurea* (EU118126) were downloaded from the NCBI database.

### Cp genome sequencing, assembly, and annotation

The total DNA was extracted from the stem samples collected from the three species using a modified CTAB method [47]. Pair-end sequencing (insert size: 350 bp) was then performed using an Illumina NovaSeq 6000 platform (Illumina, San Diego, CA, USA). Raw paired-end reads of 150 bp were processed using SOAPnuke (version 1.5.2) to remove adapters and low‐quality sequences (the unknown base ratio was higher than 5% and the low‐quality base ratio [Q < = 5] was more than 20%) [48]. The raw reads were filtered to obtain high-quality clean data. The cp genome was assembled using NOVOPlasty (version 3.7.2) [49]. *C. exaltata* was used as the reference sequence. The other setting was default (K-mer = 39). The genes in the cp genomes of *C. epithymun*, *C. europaea*, *C. chinensis*, *C. japonica* and *C. gronovii* were annotated using GeSeq software. The start and stop codons of the gene were identified using automated tools. Circular maps of the cp genomes were obtained using OGDRAW (https://chlorobox.mpimp-golm.mpg.de/OGDraw. html) [50].

### Phylogenetic analysis

Molecular phylogenetic trees were constructed using the whole cp genomes and all the protein-coding sequences of the 23 *Cuscuta* species. *Ipomoea purpurea* was used as an outgroup [29]. A total of 27 cp genomes were aligned using MAFFT v.7.450 [51] and manually adjusted using Geneious Prime 2021.1.1 (Biomatters, Ltd., Auckland, New Zealand). The maximum likelihood (ML) analysis was performed using 1,000 bootstrap replicates after selecting the best-fit substitution model via MEGA 7 [52].

### Cp genome comparison and SSR searching

GENEIOUS software was used to determine the GC content. MAVUE was used to align the cp genome and identify inversions [53]. MISA software was used to detect the SSRs in the cp genome using the following parameters: minimum SSR motif length of 10 bp and repeat times of mono-10, di-6, tri-5, tetra-5, penta-5, and hexa-5 [54].

## Conclusions

In this study, the cp genomes of *C. epithymum*, *C. europaea C. gronovii*, *C. chinensis* and *C. japonica* were sequenced and assembled. We analyzed the cp genomes of five *Cuscuta* species and compared them with the previously released cp genomes of 21 *Cuscuta* species. The complete cp genome sequences of *C. epithymum* and *C. europaea* were 96,292 and 97,661 bp long, respectively, and lacked an inverted repeat (IR) region. Based on the cp genome structure and genes loss events, we divided the 23 *Cuscuta* species into four subgenera (*Monogynella*, *Pachystigma*, *Cuscuta*, and *Grammica*). We found that *C. epithymum* and *C. europaea* belonged to subgenus *Cuscuta* for the lack of one IR region and the presence of two inversions. Furthermore, the 23 *Cuscuta* species had substantial variations in the length of their cp genome and its gene composition. Most of the reduced cp genomes lost several photosynthetic genes (*ndh*, *rpo*, *psa*, *psb*, *pet*, and *rbcL*), thus, gradually decreasing their photosynthetic capacity. This study will guide future comparative genomic investigation into the evolution of *Cuscuta* species.

## Electronic supplementary material

Below is the link to the electronic supplementary material.

Supplementary Material 1

Supplementary Material 2

Supplementary Material 3

## Data Availability

Cp genome data of five *Cuscuta* species were deposited in the NCBI database (OL752638-OL752640, OP620588, OP620589).

## Declarations

### Statement

Our experimental research and field studies on plants comply with relevant institutional, national, and international guidelines and legislation.
