# Supplementary figures and images for: Complete chloroplast genomes of five Cuscuta species and their evolutionary significance in the Cuscuta genus
Source: BMC Genomics. 2023 Jun 8;24:310. doi: 10.1186/s12864-023-09427-w (PMC10251547; doi:10.1186/s12864-023-09427-w)

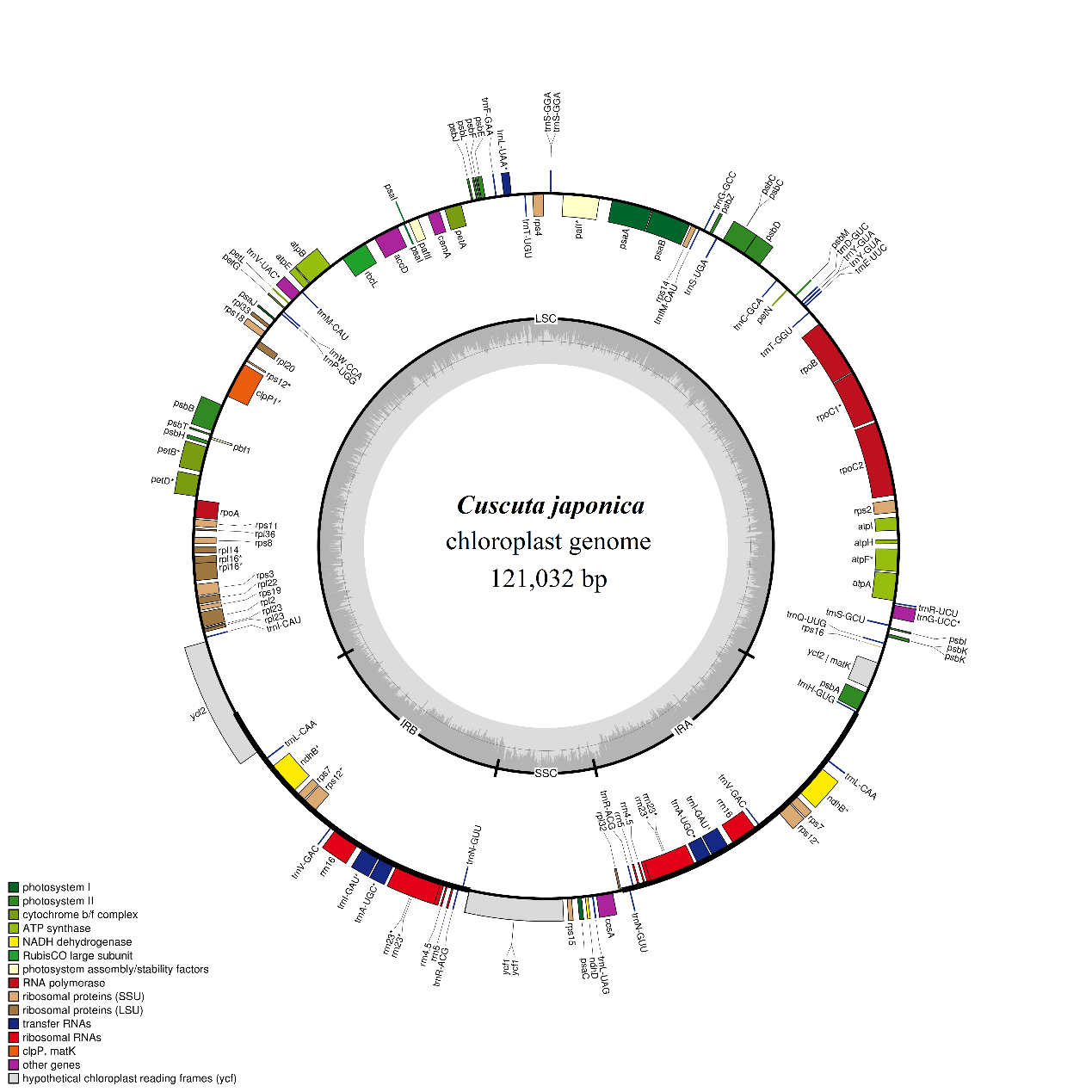


Figure S1: Genetic map of the chloroplast genomes of *Cuscuta japonica.*

Supplement: Supplementary file 1 — Supplementary Material 1 [file 12864_2023_9427_MOESM1_ESM.docx]

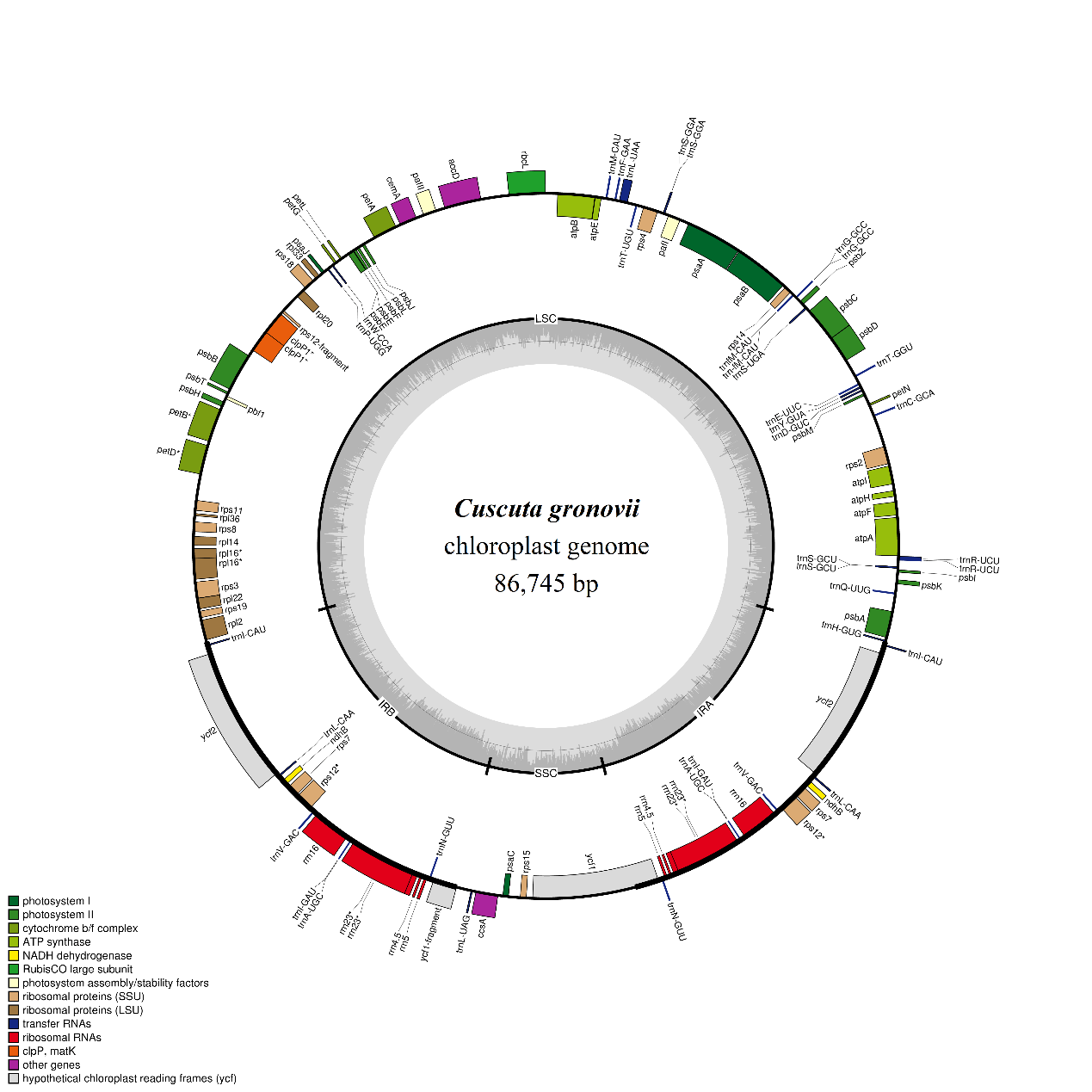


Figure S2. Genetic map of the chloroplast genomes of *Cuscuta gronovii*.

Supplement: Supplementary file 2 — Supplementary Material 2 [file 12864_2023_9427_MOESM2_ESM.docx]

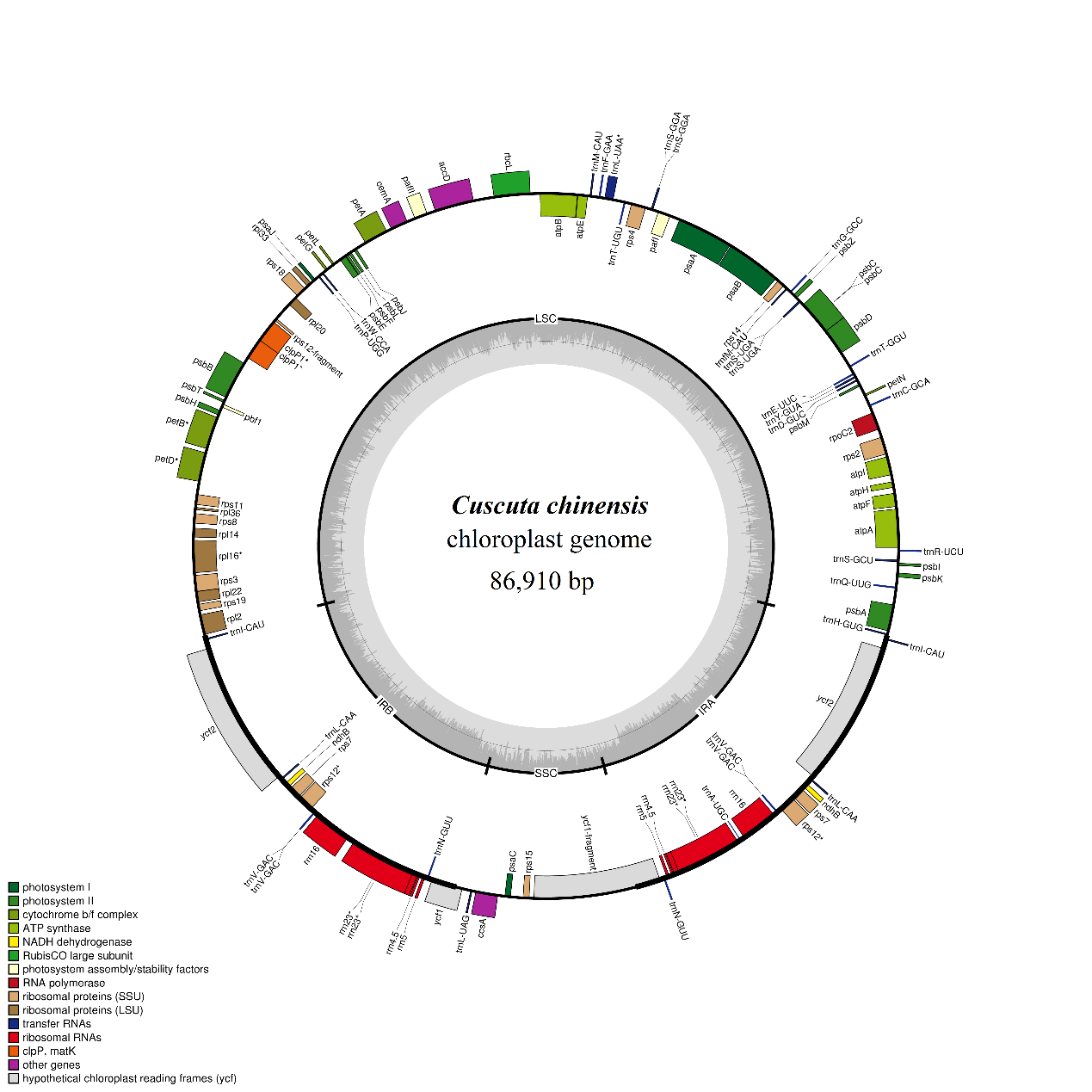


Figure S3. Genetic map of the chloroplast genomes of *Cuscuta chinensis*.

Supplement: Supplementary file 3 — Supplementary Material 3 [file 12864_2023_9427_MOESM3_ESM.docx]
